# Youth development in India: does poverty matter?

**DOI:** 10.1186/s40064-015-1410-z

**Published:** 2015-10-15

**Authors:** Bijaya Kumar Malik

**Affiliations:** Department of Education in Social Sciences, National Council of Educational Research and Training, New Delhi, India

**Keywords:** Youth development, Adolescence, Wealth index, Healthy lifestyle, Poverty

## Abstract

This paper explores the differentials in youth development patterns determined by the economic condition of the household in India. The wealth index is used to glean youth development differentials in the different economic categories of the household. The findings suggest that youth from the bottom 20 per cent (poorest) of households are deprived in education, employment, labour force and are not working currently compared to youth from the middle and rich households. The states differ in youth development patterns (employment, appropriate education, skill development and awareness about health). There are more working youth among poor households than among rich households in India. Female youth are more disadvantaged compared to male youth and it is the same with the rural–urban distribution of youth. This paper concludes that the various economic categories/wealth index (poorest, poorer, middle, richer and richest) directly determine the pattern of youth development in India.

## Background

India has one of the highest adolescent (253 million) and youth populations in the world. The Census of India (2011) has highlighted the profile and status of the adolescent and youth population, which constitutes a critical segment of the total population of India. Socio-political, economic and demographic developments depend on them. The transition from education and training to economic activity marks an important phase in the lives of youth, who are the productive workforce of the country. The huge unemployment among youth due to lack of skills and poverty is a long term challenge for India.

In 2010, it was estimated that the population of the world was 6.91 billion and that adolescent population (10–19 years) constituted 1.19 billion and youth (15–24 years) 1.22 billion, which together accounted for 26.3 per cent of the total population of the world (World Population Prospects: the 2012 Revision, June 2013). In India, the adolescent population is 253 million and the youth population is 231.9 million, which constitute 20.9 per cent and 19.2 per cent of the total population respectively and both adolescent and youth population comprise 40.1 per cent of the total population of India (Census of India 2011). Compared to the 2001 Census, the percentage of adolescents has declined, while that of youth has increased due to a decline in the level of fertility. There was an addition of nearly 181.9 million to India’s total population during the 2001–2011 Census and youth population added 41.8 million to its population segment. The youth population of India is so huge that it is equivalent to the total population of eighteen countries in western Asia according to United Nations estimates (World Population Prospects: The 2012 Revision 2013).

Youth is defined as those persons in the age group 15–24 years by the United Nations, though the age range for youth may vary in different countries due to different contexts and needs of youth. During this transitional phase, physical, educational, psychological, social and economic changes occur in their lives. The India National Youth Policy (NYP) covers all youth in the age group 13–35 years, which is divided into two groups, that is, 13–19 years and 20–35 years (National Youth Policy 2003). The recent National Youth Policy has defined youth as those in the age group 15–29 years, who comprise 27.5 per cent of the population. Youth is a more fluid category than a fixed age-group. ‘Youth’ is often indicated as a person between the age where she/he leaves compulsory education, and the age at which she/he finds her/his first employment (National Youth Policy 2014). The study, *Youth in India: Situation and Needs*, considered youth as those in the age group 15–24 years and this paper follows this definition.

Every year, the Government of India allotted Rs. 2710/-per youth per year for development in terms of employment, appropriate education, skill development and awareness about health (Union Budget, 2011–2012). State governments, institutions, other stakeholders and Non-Governmental Organizations (NGOs) also supported the development of youth, towards making them a productive workforce.

In 2000, the United Nations Millennium Development Goals (MDGs) committed to combat HIV/AIDS, malaria and other diseases under Goal 6: target 19, that is, equipping those in the age group, 15–24 years with comprehensive and correct knowledge of HIV and AIDS and evolving a global partnership for development under Goal-8: target-45, that is, unemployment rate of young people aged 15–24 by sex by 2015 for all countries Youth seemed to have heard about these issues, but lacked comprehensive knowledge.

### Importance of youth for the demographic dividend in the Indian context

In many countries, demographic transition is achieved after the large segment of adolescent and youth population joins the total population. This happens only when there is a transition of its population from a high to a low situation for both mortality and fertility over a particular period, which also known as the demographic window of opportunity. Demographic dividend can be achieved when economic growth accelerates. This occurs when the working age group population, having acquired technical and vocational skills, engage themselves in economic activities. The implementation of national policies over a period of time supports the process. This significant shifting of age structure in the Indian population, can increase economic participation and reduce dependency, which will support economic growth. Many demographers and economists have forecast that India will reap the demographic dividend through its working population, which has a huge latent potential and productivity. Literacy rate among youth increased from 36 per cent in 1962 to 86.1 per cent in 2011. There is some difference between male literacy (90 %) and female literacy (81.8 %), and that of rural youth (83.7 %) and urban youth (91.4 %) youth according to Census, 2011.

## Review of literature

Various research studies have shown how socioeconomic factors determine the youth development pattern in the Indian context. There is evidence that the young (16–24 years) are particularly more prone towards the negative effects of recession, which create a spell of unemployment (Bell and Blanchflower 2010). Global recession creates a huge volume of temporary employment among them (Higgins and Niall’ 2012). Low literacy rate and health problems among female youth are obstacles for the development of youth in India (Dreze et al. 2011).

### Rationale for the study

The youth population in any country is dynamic and important for its long run development. The latent power and demographic shift of the Indian youth population can improve our economy. In 2014, the Government of India formulated a National Youth Policy covering eleven priority areas—*Education, Employment and Skill Development, Entrepreneurship, Health and Healthy Lifestyle, Sports, Promotion of Social Values, Community Engagement, Participation in Politics and Governance, Youth Engagement, Inclusion, and Social Justice*—which provides youth a strong road map for realizing the proposed goals during the next 5 years with an appropriate framework. NYP (2014) aims to empower Indian youth to utilize their full potential. According to this policy, youth in the age group, 15–29 years comprises 27.5 per cent of the population. This significant segment of population can increase its labour participation and productivity to better our economy. It is estimated to contribute about 34 per cent of the Gross National Income (NYP 2014).

The Census of India (2011) has released a number of indicators on youth including other age groups, literacy, work status, total population and age wise population. Some important socioeconomic and demographic indicators are to be released by the Census, which will help researchers and academicians to investigate youth development in detail for formulating national plans and policies. Socioeconomic and demographic variables are not available from the Census of India currently; despite these constraints, this research paper makes an effort to study how various factors especially poverty/wealth index is related to the youth development pattern (employment, appropriate education, skill development and awareness about health) in India by using data from the demographic survey, *Youth in India: Situation and Needs: 2006*–*2007* conducted by IIPS, Mumbai and Population Council, which covered key areas like education, unemployment, work participation rate and other demographic variables. In this paper, wealth index and other related variables have been used as background variables to know the differentials of youth engagement and their developmental pattern in India.

## Research questions

1. Does poverty determine the pattern of youth development (employment, appropriate education, skill development and awareness about health) in India?
2. What are the social, cultural and other barriers to youth development in India?
3. What kind of national policy framework will provide more support and empower youth in India?

## Objectives

The broad objective of this research paper is to understand the role of poverty in youth engagement/employment pattern in India. The specific objectives are

1. To examine the pattern of youth development (employment, appropriate education, skill development and awareness about health) differentials linked with poverty in India.
2. To know the extent of youth economic engagement in the development of India and its States.

## Data and methodology

The data for this paper is derived from *Youth in India Situation and Needs Study,* which was conducted by the International Institute for Population Sciences (IIPS), Mumbai and Population Council, New Delhi, in 2006–2007. It covers six states, Rajasthan, Bihar, Jharkhand, Maharashtra, Andhra Pradesh (erstwhile) and Tamil Nadu reflecting the diversity and geographic coverage of India. This study covers 174,037 households and 50,848 young people (15–24 age group). The main domain of this data set covers a wide range of issues on young people’s *livelihood, education, family life education, sex and sexuality, adolescence education dynamics*. This study is best suited for this research as all of these young people were adolescents 6 years ago and recent behaviour and economic participation issues can be explored.

Information on youth development in socioeconomic and demographic areas in India is not sufficient and systematic. However, this study is unique in gathering information on youth development (employment, appropriate education, skill development and awareness about health) and exploring its sociodemographic determinants in these six States. This research paper adopted some statistical techniques such as bivariate and multivariate analysis, and logistic regression. Apart from this secondary data set, I have linked youth related issues with data from Census 2011. The term used in this paper as employment (currently those who are working), un-employment(those who are actively searching employment are not getting at existing wage rate) and labor force (currently working and same time unemployed).

## Findings and discussions

In Table 1, the percentage of various age groups to the total age group has been estimated from Census 2011. The adolescent age group (10–19 years) and youth age group (15–24 years) form a significant section of the total population of India. India can realize the demographic dividend by enabling and empowering more youth through targeted areas such as skill development, appropriate education, healthy lifestyle and non targeted areas such as food subsidies and employment opportunities.

**Table 1:** Percentage of various age groups in India, 2011

S. no.	Age group	% of various age groups to the total population
1	0–4	9.32
2	5–9	10.48
3	10–14	10.96
4	15–19	9.95
5	20–24	9.20
6	25–39	22.72
7	40–59	18.42
8	60–79	7.65
9	80+	0.93
10	Age not stated	0.37

Source: Estimated from Census age wise final data, 2011

In Table 2, the work participation rate among youth is explained by analyzing the data of Census 2011. There is a significant differential in work participation, among various youth categories—the age group, 15–19 years, indicates 25.5 per cent compared to 49.8 per cent in the age group, 20–24 years. In the age group 15–24 years, the work participation is 36.9 per cent, compared to 39.8 per cent among all ages. Gender differentials in work participation are significant, that is, for male youth, it is 47.5 per cent, while for female youth, it is only 25.4 per cent. This indicates that women work participation has to increase considerably for their development. A country will realize its demographic dividend when both male and female youth development in terms of higher education, work participation, skill development and healthy lifestyle is achieved equally. Rural youth have better (41.6 %) work participation compared to their urban counterparts (27.1 %). This may be because urban youth (15–24 years) concentrate more on higher education and have an urban lifestyle.

**Table 2:** Total work participation rate among youth in India 2011

Youth categories	Total	Male	Female	Rural	Urban
15–19	25.1	30.8	18.6	28.9	16.4
20–24	49.8	66.0	32.4	56.1	37.5
15–24	36.9	47.5	25.4	41.6	27.1
All ages	39.8	53.3	25.5	41.8	11.0

Source: RGI and UNFPA, adolescents and youth profile in India, 2011 (based on Census 2011)

Work participation rate trend and differentials among youth in India from 1981 to 2011 Census is discussed in Table 3, which shows that the work participation rate among youth (15–24 years) has decreased for total, male female, rural and urban respectively. In 1981, total youth work participation rate was 47.1 per cent, which reduced to 36.9 per cent in 2011 and this trend was found in all categories. Rural youth work participation seems to be reducing more when compared to the others. Current educational opportunities offered to youth seem to lead towards youth development on the whole.

**Table 3:** Work participation rate trend and differentials among youth in India (1981–2011)

Census year	Total	Male	Female	Rural	Urban
1981	47.1	65.1	27.8	53.5	30
1991	44.6	58.2	29.8	51.3	27.7
2001	42.4	53.6	30	49.3	26.9
2011	36.9	47.5	25.4	41.6	27.1

Source: RGI and UNFPA, adolescents and youth profile in India, 2011 (based on Census, 2011)

**Fig. 1:**
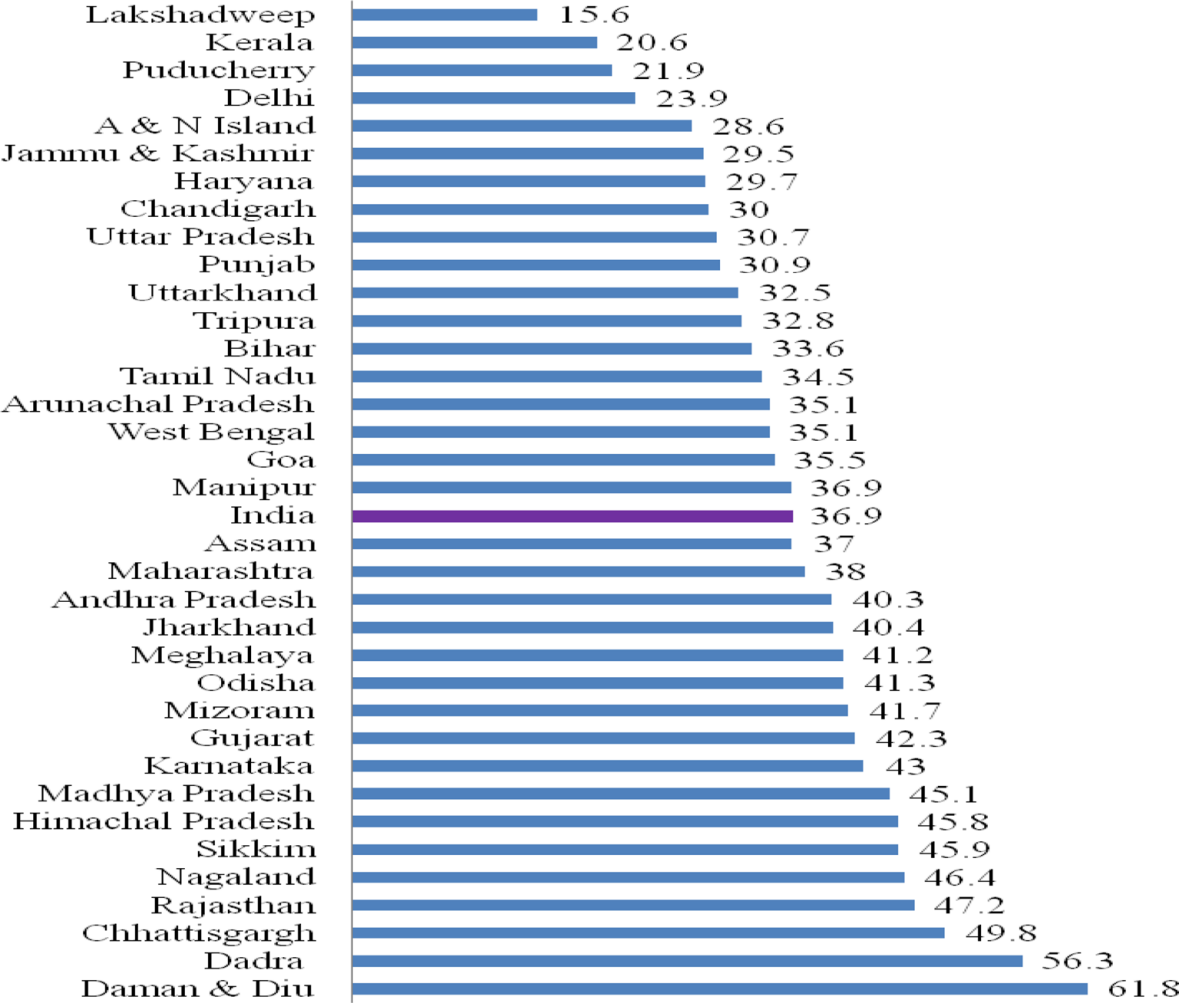
Work participation rate among youth in states/UTs of India, 2011. Source: Registrar General of India (RGI )and UNFPA, Adolescents and youth profile in India, 2011 ( Based on Census,2011) (include ‘erstwhile’ before Andhra Pradesh)

Figure 1 shows the work participation rate among the youth in the States/Union Territories (UTs) of India. The figure indicates that the work participation among youth in small UTs like Daman and Diu is the highest (61.8 %) followed by Dadra Nagar Haveli (56.3 %) and the lowest (15.6 percent) is in Lakshadweep followed by Kerala (20.6 %). The national average of youth work participation is 36.9 per cent. States/UTs with high youth work participation, above the national average, are (erstwhile) Andhra Pradesh, Assam, Maharashtra, Jharkhand, Meghalaya, Odisha, Mizoram, Karnataka, Gujarat, Madhya Pradesh, Himachal Pradesh, Sikkim, Nagaland, Rajasthan, Chhattisgarh and below national average are Puducherry, Andaman and Nicobar Islands, Jammu and Kashmir, Haryana, Chandigarh, Uttar Pradesh, Punjab, Uttarakhand, Tripura, Bihar, Tamil Nadu, Arunachal, West Bengal, Goa and Manipur.

In Table 4, the percentage of youth employment by wealth index in six states has been presented. The finding clearly indicates that fewer youth (13 %) from the poorest of the poor (quintile-1) households are employed compared to youth from the richest (quintile-5) households (22.3 %) in total for the six states under study. This finding shows that more youth in the age group (15–24 years) from the poorer strata of society are unemployed. This need for unemployment, perhaps, is a reflection of their poor economic condition, lack of monetary resources to fund education or familial insistence. Poverty has a great impact on youth development in every quintile, in all the six states. There are some differentials in negative youth development, which affect youth development due their employment at an early age and lack of proper education. These factors make them feel insecure.

**Table 4:** Percentage of youth employment by wealth index in six states

Name of state	Wealth index (quintile)					
	Poorer	Poor	Middle	Richer	Richest	Total
Rajasthan	3.4	4.2	3.3	7.3	9.8	6.0
Bihar	21.7	21.5	36.4	40.1	53.5	28.2
Jharkhand	15.1	17.6	27.9	33.9	44.1	23.0
Maharashtra	8.3	12.5	15.0	23.1	27.8	18.0
Andhra Pradesh (erstwhile)	4.9	6.7	6.6	9.7	14.9	8.5
Tamil Nadu	7.9	8.0	9.2	10.3	18.0	10.9
Total	13.0	12.0	13.1	16.1	22.3	15.0

Table 5 presents the percentage of youth are not in labour force by wealth index in the six States under study. The findings indicate that there are fewer youth from the poorest of the poor (quintile-1) households (56.2 %) are not in the labour force compared to youth from the richest (quintile-5) households (71.9 %) in the six states. All the six states in this study show the same trend. This finding shows that more youth in the age group, 15–24 years from the poorer strata of society are not in the labour force. In other words, it indicates that due to familial constraints and their poor economic condition, they are not in the labour force. Poverty has a negative impact on youth development in every quintile in all the six states.

**Table 5:** Percentage of youth are not in labour force by wealth index in six states

Name of state	Wealth index (quintile)					
	Poorer	Poor	Middle	Richer	Richest	Total
Rajasthan	49.4	53.3	56.9	59.8	69.7	60.2
Bihar	63.9	67.5	65.2	65.6	72.6	66.1
Jharkhand	68.2	68.5	74.1	75.0	77.7	71.5
Maharashtra	40.0	51.5	56.3	62.6	71.0	59.6
Andhra Pradesh (erstwhile)	40.0	46.4	53.1	55.7	69.0	55.2
Tamil Nadu	61.3	57.5	59.1	66.5	76.7	66.0
Total	56.2	58.2	58.4	61.8	71.9	61.8

Table 6 shows the percentage of youth currently not working by wealth index in these six States. The finding clearly indicates that fewer youth (36.4 %) from the poorest of the poor households (quintile-1) are currently not working compared to youth (70.4 %) from the richest households (quintile-5) in these six States. This trend among the richest and the poorest youth is the same for all these six these States. This finding shows that more youth in the age group, 15–24 years from the poorer strata of society are currently working. This reflects that poverty and economic restraints have prevented these youth from acquiring further education, which affects their development. In other words, it indicates, they are not in higher education or due to their bad economic condition, they are force to work currently in any form for them and their family. Poverty has an great impact in youth development found in this all six states as in every quintiles, there is some differentials in youth development in negative condition. Within states also similar trend. This will affect the youth development due their current working condition in this earlier age and not getting the appropriate education and healthy condition for their healthy lifestyle.

**Table 6:** Percentage of youth currently not working by wealth index in six states

Name of state	Wealth index (quintile)					
	Poorer	Poor	Middle	Richer	Richest	Total
Rajasthan	30.1	29.8	37.2	46.8	63.8	45.7
Bihar	42.6	47.6	55.4	63.4	74.9	51.1
Jharkhand	33.3	38.0	53.9	65.6	77.6	48.1
Maharashtra	29.8	41.1	49.2	61.9	73.3	56.1
Andhra Pradesh (erstwhile)	27.1	33.7	39.7	45.5	63.5	44.5
Tamil Nadu	42.4	43.1	48.9	60.2	75.4	58.4
Total	36.4	40.3	46.4	55.3	70.4	51.2

The percentage of youth who are employment, in the labour force and currently not working by gender and wealth index has been explained in the above Table 7. The findings show that fewer youth from the poorest households are employed (13 %), are in the labour force (56.1 %) and currently not working (36.4 %) compared to youth from the richest households (22.3 %), who are in the labour force (71.9 %) and currently not working (70.4 %) in total for both genders. The trend is similar for both male and female youth. In all these three economic parameters, females are at a disadvantage—total employment for male youth is 16 per cent compared to 14.1 per cent for female youth; 71.1 percent of male youth are in the labour force compared to 39.7 per cent for female youth, and 59.9 per cent of male youth are currently not working compared to 32.2 per cent of female youth. Female youth are less in employment, in labour force and currently working compared to male youth in all five ladders or economic conditions (Q-1, Q-2,Q-3, Q-4 and Q-5) respectively. It clearly shows that poor female youth in India are in a worse situation due to their deprivation. Poverty and other social factors contribute to their lagging behind. This female youth mass is a big segment of the Indian population. It is only when they are given appropriate education and equipped with skills and a healthy lifestyle that Indian youth can reap the demographic dividend of our country. These issues have been emphasized by the recent National Youth policy (2014) framework by the Government of India, Ministry of Youth Affairs and Sports.

**Table 7:** Percentage of Youth employment, in labour force and currently not working by gender and wealth index

Youth development pattern by sex	Wealth Index (quintile)					
	Poorest	Poor	Middle	Richer	Richest	Total
Employment						
Male	13.4	12.2	13.0	14.2	17.9	16.0
Female	12.	11.8	13.1	18.5	27.6	14.1
Total	13.0	12.0	13.1	16.1	22.3	15.0
In labour force						
Male	33.2	35.9	35.3	37.6	52.5	72.1
Female	64.0	68.8	69.2	74.5	81.2	39.7
Total	56.2	58.2	58.4	61.8	71.9	61.8
Currently not working						
Male	19.1	21.9	27.0	33.1	51.4	59.9
Female	42.2	49.1	55.4	66.9	79.5	32.2
Total	36.4	40.3	46.4	55.3	70.4	51.2

The percentage of youth employed, in the labour force and currently not working by rural, urban with wealth index has been explained in the above Table 8. Findings show that fewer youth from the poorest households are employed (13 %), in the labour force (36.4 %) and currently not working (36.4 %) compared to youth from the richest households, who are higher in employment (22.3 %), in the labour force (70.4 %) and currently not working (70.4 %) in total for both rural and urban areas respectively. There are similar trends for both rural and urban youth in these six states. In all these three economic parameters rural youth are at a disadvantage of 14.4 per cent of rural male youth are employed compared to 16.9 per cent of urban youth, 44.5 per cent of rural youth are in the labour force compared to 66.8 per cent of urban youth and 44.5 per cent of rural youth are currently not working compared to 66.8 per cent of urban youth. Rural poor youth are less in employment, in labour force and currently not working compared to urban youth in all five ladders or economic conditions (Q-1, Q-2,Q-3, Q-4 and Q-5) respectively. Rural youth comprise 18.9 per cent of the population, while urban youth comprise 19.7 per cent of the population according to Census 2011. It is therefore necessary to focus on equipping rural youth with appropriate education and skills so as to foster youth development in India.

**Table 8:** Percentage of youth employment, in labour force and currently not working by rural and urban with Wealth Index

	Wealth Index (quintile)					
	Poorest	Poor	Middle	Richer	Richest	Total
Employment						
Rural	10.2	12.7	11.8	16.0	22.0	14.4
Urban	13.1	11.9	13.4	16.1	22.6	16.9
Total	13.0	12.0	13.1	16.1	22.3	15.0
In labour force						
Rural	54.6	53.3	56.5	64.1	74.8	44.5
Urban	35.2	38.9	43.6	49.8	63.5	66.8
Total	36.4	40.3	46.4	55.3	70.4	51.2
Currently not working						
Rural	54.6	53.3	56.5	64.1	74.8	44.5
Urban	35.2	38.9	43.6	49.8	63.5	66.8
Total	36.4	40.3	46.4	55.3	70.4	51.2

In the above Table 9, the percentage of youth employed by their education level has been explained. Findings show the levels of employment for youth with less than 5 years of schooling in Bihar (14.9 %) compared to those from the erstwhile State of Andhra Pradesh (.8 %). It clearly shows that at the same education level, youth are at a greater disadvantage in Andhra Pradesh compared to those in Bihar. Andhra Pradesh youth should promote more youth development programmes, especially for youth with lower levels of education. Among youth with higher levels of educational (13 years of schooling and above) Jharkhand youth have a higher employment percentage (42.5 %) compared to those from erstwhile Andhra Pradesh (9.8 %). Findings also show that youth in Rajasthan have higher levels of education than those in other all the other states and that youth from Jharkhand are the best among all these six states for total categories.

**Table 9:** Percentage of youth employment by their educational level in six states

Name of states	Education (years of schooling)				Total
	Lt5	5–9	10–12	13+	
Rajasthan	3.5	5.3	12.1	5.5	6.0
Bihar	14.9	32.3	56.8	16.6	28.2
Jharkhand	11.1	21.7	48.1	42.5	23.0
Maharashtra	2.9	9.9	31.2	39.7	18.0
Andhra Pradesh (erstwhile)	0.8	2.8	19.7	9.8	8.5
Tamil Nadu	2.3	4.9	18.8	26.1	10.9
Total	5.7	10.0	29.6	16.3	15.0

In the above Table 10, the percentage of youth currently not working by their educational level has been explained. There are no big differentials in youth currently not working. One important finding is that the number of less educated youth currently working from all these six states is higher compared to highly educated youth. Youth who are not educated and are compelled to work in unhealthy surroundings, are deprived of higher education and a healthy lifestyle. Education determines positive youth development in these six States.

**Table 10:** Percentage of youth currently not working by their education and wealth index in six states

Name of states	Education (years of schooling of youth)				Total
	Lt5	5–9	10–12	13+	
Rajasthan	30.5	48.9	70.5	34.1	45.7
Bihar	43.5	59.0	59.3	44.1	51.1
Jharkhand	34.7	52.5	69.2	60.2	48.1
Maharashtra	31.9	54.5	66.0	62.4	56.1
Andhra Pradesh	23.9	41.2	57.5	40.1	44.5
Tamil Nadu	38.4	47.9	71.6	68.3	58.4
Total	34.3	50.9	64.9	45.7	51.2

In Table 11, the percentage of youth employed by their father’s education and poverty have been explained. Analysis shows that the father’s educational level has an impact on youth development in general. Youth from illiterate father are less (8.8 %) employed compared to youth those fathers with 12 years of education and above (35.9 %) under total and it is same trend for all poverty level. This indicates that the higher the father’s level of education, the greater the employment for the son/daughter. The five categories of wealth quintiles also indicate the same pattern. So, the father’s educational level is also an important factor of youth development in India.

**Table 11:** Percentage of youth employment by father’s education and poverty status in india

Education of Father	Wealth index (quintile)					
	Poorer	Poor	Middle	Richer	Richest	Total
Non-literate, literate with no formal schooling	10.2	7.4	7.4	10.4	9.0	8.8
1–7 years of schooling	12.2	12.3	14.7	15.6	16.0	14.3
8–11 years of schooling	27.9	27.2	21.6	22.0	26.2	24.4
12 and above years of schooling	28.6	37.1	39.4	36.2	35.4	35.9
Don’t know/missing	21.3	10.2	14.1	15.1	19.8	15.4
Total	13.0	12.0	13.1	16.1	22.3	15.0

In Table 12, the percentage of youth employment by their father’s education and poverty in six states has been presented. Analysis shows that the father’s educational level has an impact on youth development in general. Youth from illiterate father are less (8.8 %) employed compared to youth those fathers with twelve years of education and above (35.9 %) under total and it is same trend for all poverty level. It clearly shows that more sons/daughters of illiterate fathers are employed compared to those whose fathers with higher levels of education. Five categories of quintiles and all six states also indicate the same pattern. Thus, the father’s educational level is an important factor of youth development in these six states.

**Table 12:** Percentage of youth employment by father’s education and wealth index in six states

Name of state	Wealth index (Quintile)					
	1st Quintile	2nd Quintile	3rd Quintile	4th Quintile	5th Quintile	Total
Rajasthan						
Illiterate	3.7	3.2	3.3	5.2	3.0	3.7
1–7 years of schooling	3.9	8.8	2.7	6.7	8.3	6.4
8–11 years of schooling	0.0	7.4	3.9	11.3	12.5	9.9
12 and above years of schooling	0.0	7.8	9.9	22.3	18.4	17.2
Don’t know/missing	0.0	0.0	0.0	7.6	14.5	5.4
Total	3.4	4.2	3.3	7.3	9.8	6.0
Bihar						
Illiterate	16.2	13.5	26.5	46.4	46.6	18.7
1–7 years of schooling	21.6	19.1	36.8	29.4	6.6	23.5
8–11 years of schooling	42.3	39.9	38.4	29.9	53.1	39.8
12 and above years of schooling	36.3	44.5	81.6	68.9	72.3	63.1
Don’t know/missing	32.0	14.5	31.1	38.1	29.7	25.9
Total	21.7	21.5	36.4	40.1	53.5	28.2
Jharkhand						
Illiterate	11.7	12.9	21.0	33.1	43.1	14.9
1–7 years of schooling	22.1	19.1	30.1	32.7	34.5	24.9
8–11 years of schooling	28.5	28.1	31.9	32.1	41.0	32.8
12 and above years of schooling	89.5	21.6	37.7	41.2	56.4	48.4
Don’t know/missing	13.9	17.9	33.1	33.9	18.7	20.8
Total	15.1	17.6	27.9	33.9	44.1	23.0
Maharashtra						
Illiterate	7.9	8.4	6.1	18.1	15.9	9.8
1–7 years of schooling	8.9	12.0	18.8	21.6	22.7	17.1
8–11 years of schooling	7.9	18.5	18.9	26.3	31.1	24.9
12 and above years of schooling	20.4	41.4	33.2	31.6	30.1	31.2
Don’t know/missing	0.0	11.3	7.5	33.3	31.4	17.2
Total	8.3	12.5	15.0	23.1	27.8	18.0
Tamil Nadu						
Illiterate	6.2	5.0	6.4	9.5	12.4	7.2
1–7 years of schooling	5.0	7.3	8.6	8.9	15.4	9.4
8–11 years of schooling	14.4	20.7	14.3	14.2	21.6	17.5
12 and above years of schooling	0.0	0.0	21.3	0.0	17.4	13.9
Don’t know/missing	39.6	0.0	14.1	9.3	9.2	10.3
Total	7.9	8.0	9.2	10.3	18.0	10.9
Total for all six states						
Illiterate	10.2	7.4	7.4	10.4	9.0	8.8
1–7 years of schooling	12.2	12.3	14.7	15.6	16.0	14.3
8–11 years of schooling	27.9	27.2	21.6	22.0	26.2	24.4
12 and above years of schooling	28.6	37.1	39.4	36.2	35.4	35.9
Don’t know/missing	21.3	10.2	14.1	15.1	19.8	15.4
Total	13.0	12.0	13.1	16.1	22.3	15.0

To understand the determinants of youth unemployment, a logistic regression has been carried out as shown in Table 13. The dependant variable is dichotomous, that is ‘1’ for youth unemployment and ‘0’ for youth employment. The independent variables are sex, age of youth, caste of respondent, religion, education of youth, education of father, standard of living and States. It is found that standard of living, education of father, type of school and education of youth are significant predictors of youth unemployment in these six states. For example, the odd of 12 years and above schooling of youth is 6.40 times higher employment rate compared to those youth have only studied 5–9 years of schooling. This indicates that more the years of schooling among the youth, less the unemployment rate in them.

**Table 13:** Logistic regression of unemployment among youth (1 = unemployment and 0 = employment)

Unemployment among youth	Exp (B)	Significance
Sex		
Men (R)	1	
Women	1.55	0.00
Age of youth		
15–19 years (R)	1	
20–24 years	0.77	0.00
Caste of the respondent		
Scheduled caste (R)	1	0.00
Scheduled tribe	1.02	0.00
Other backward caste	0.83	0.00
General	0.79	0.00
Don’t know/no caste	0.39	0.00
Religion		
Hindu (R)	1	
Muslim	1.10	0.00
Others	0.97	0.00
Education of youth		
Less than 5 years of schooling (R)	1	
5–9 years of schooling	1.95	0.00
10–12 years of schooling	6.40	0.00
13 and above years of schooling	3.21	0.00
Education of father		
Non-literate, literate with no formal schooling (R)	1	
1–7 years of schooling	1.51	0.00
8–11 years of schooling	2.16	0.00
12 and above years of schooling	3.25	0.00
Don’t know	1.59	0.00
Standard of living		
1st Quintile (R)	1	0.00
2nd Quintile	1.11	0.00
3rd Quintile	1.41	0.00
4th Quintile	1.79	0.00
5th Quintile	1.94	0.00
States		
Rajasthan (R)	1	0.00
Bihar	7.37	0.00
Jharkhand	7.63	0.00
Maharashtra	3.35	0.00
Andhra Pradesh (erstwhile)	1.49	0.00
Tamil Nadu	1.77	0.00
Constant	0.00	0.00

## Conclusion

The findings suggest that wealth index or standard of living (SLI) directly influences and determines youth development in India. Youth from the poorest households (quintile-1) are in the labour force and are more deprived or unemployed compared to youth from the richest households (quintile-5) and also those from the other three quintiles/economic levels of households in these six states. The father’s education and education of youth is the second pillar of youth development in India, which is influenced by the educational level of both. The higher the education of the father, the lesser the number of youth working in the labour force. These six States have differ in the patterns of youth development. Moreover, rural youth are more disadvantaged than urban youth, and female youth are more disadvantaged than male youth in these six states of India, irrespective of caste and region. Poverty/wealth index is an influential factor for youth development in India, which may be considered the first pillar of youth development. In every situation, the wealth index clearly shows that the lower the economic condition of the household, the more disadvantaged the youth. Poverty definitely leaves its mark on youth development in India.

## Limitations

“This paper makes an attempt to reflect about the youth development pattern in India by using the data from Youth in India: Situation and Needs (2006–2007) although it’s sample size is small for generalizing the facts for whole India”. However, this data set is pioneer in the context of youth related information in Indian context.
